# Multidisciplinary Management of Traumatic Profunda Femoris Artery Branch Hemorrhage With Coil Embolization: A Case Report

**DOI:** 10.7759/cureus.101605

**Published:** 2026-01-15

**Authors:** Susanne Kraske, Tom Allert, Robin Bülow

**Affiliations:** 1 Department of Pediatric Surgery, University Medicine Greifswald, Greifswald, DEU; 2 Department of Pediatrics, University Medicine Greifswald, Greifswald, DEU; 3 Institute for Diagnostic Radiology and Neuroradiology, University Medicine Greifswald, Greifswald, DEU

**Keywords:** blunt trauma, coil embolization, compartment syndrome, profunda femoris artery, rollover extremity injury

## Abstract

Rollover extremity trauma can result in severe injuries like open or closed fractures, neurovascular and muscle damage or decollement (soft-tissue degloving). After severe blunt trauma, the risk of compartment syndrome (CS) must also be considered. Early clinical signs of CS are pain out of proportion and increased pain with passive stretching of muscles within the affected compartment. To avoid irreversible muscle or nerve damage, early detection of CS is necessary. However, measurement of compartment pressure for detection is discussed controversially. Its reliability depends on technical skills and patient conditions like blood pressure and comorbidities.

In this article, we report the case of a 15-year-old boy who sustained a rollover injury to the thigh. We describe the multidisciplinary management, including endovascular treatment, and discuss the risk of CS following extremity trauma as well as available diagnostic approaches.

## Introduction

Compartment syndrome (CS) of the thigh is a rare condition, which could be seen in well-trained young men with high muscle mass after blunt trauma in sports, after motor vehicle collisions, in patients with arterial injury due to femur fractures, osteosynthesis or stab and shotgun wounds [1-3].

Delayed diagnosis of CS of the thigh leads to neuromuscular deficits with permanent quadriceps impairment and persistent thigh weakness [2,3]. Impairments were associated with a time to surgical decompression of greater than 8 hours [3]. 

Increasing pain and pain out of proportion, especially following passive muscle stretch, are early signs of CS. Pallor, paresthesia, paralysis, and pulselessness are late signs [2,3].

We report the case of a 15‑year‑old boy who sustained active bleeding from a branch of the profunda femoris artery following a rollover injury to his thigh. We describe the multidisciplinary care and discuss the risk of CS after extremity injury and its diagnostic possibilities.

## Case presentation

A 15-year-old boy sustained a rollover injury during a motocross tournament when he fell and a motorcycle rolled over his right thigh. The patient was initially evaluated in accordance with Advanced Trauma Life Support (ATLS) protocol in the emergency department of a nearby Level III trauma center, located 30 km from our hospital. He was hemodynamically stable, and no injuries other than blunt trauma to the right thigh were identified.

There were no abnormalities in the patient’s bleeding history. Neither the patient nor his family had any known coagulation disorders, and the standard coagulation parameters (PT/INR and PTT) were within normal limits.

Physical examination of the right thigh revealed no visible deformity, no external skin lesions and no tire‑mark impressions. The thigh was visibly swollen, with a noticeably greater circumference compared to the contralateral side. The overlying skin was warm, without signs of décollement or abnormal tension, and it did not appear shiny. Active and passive stretching of the thigh muscles elicited severe pain.

**Figure 1:**
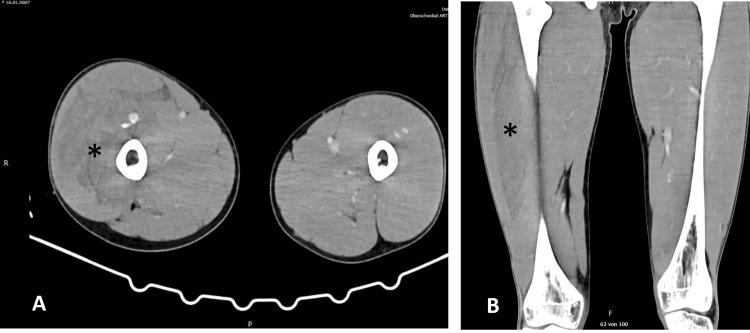
Massive swelling of the right thigh A shows the transverse angiographic CT scan of both thighs, with a clearly increased circumference of the right thigh, visible on the left side of the image. B shows a coronal angiographic CT scan of both thighs, demonstrating edema of the right thigh, appearing as hypodense striae within the quadriceps muscle (*).

Due to severe initial pain and marked swelling of the right thigh, computed tomography (CT) angiography was performed. Imaging revealed active bleeding from a deep branch of the profunda femoris artery and a spindle-shaped hematoma within the medial and anterior compartments of the right thigh, measuring 9.0 × 4.5 cm with a maximal longitudinal extension of 27 cm (Figure 1). The patient was subsequently referred to our Level I trauma center. In the meantime, radiological images were transmitted via the picture archiving and communication system (PACS) to facilitate immediate interdisciplinary treatment planning.

**Figure 2:**
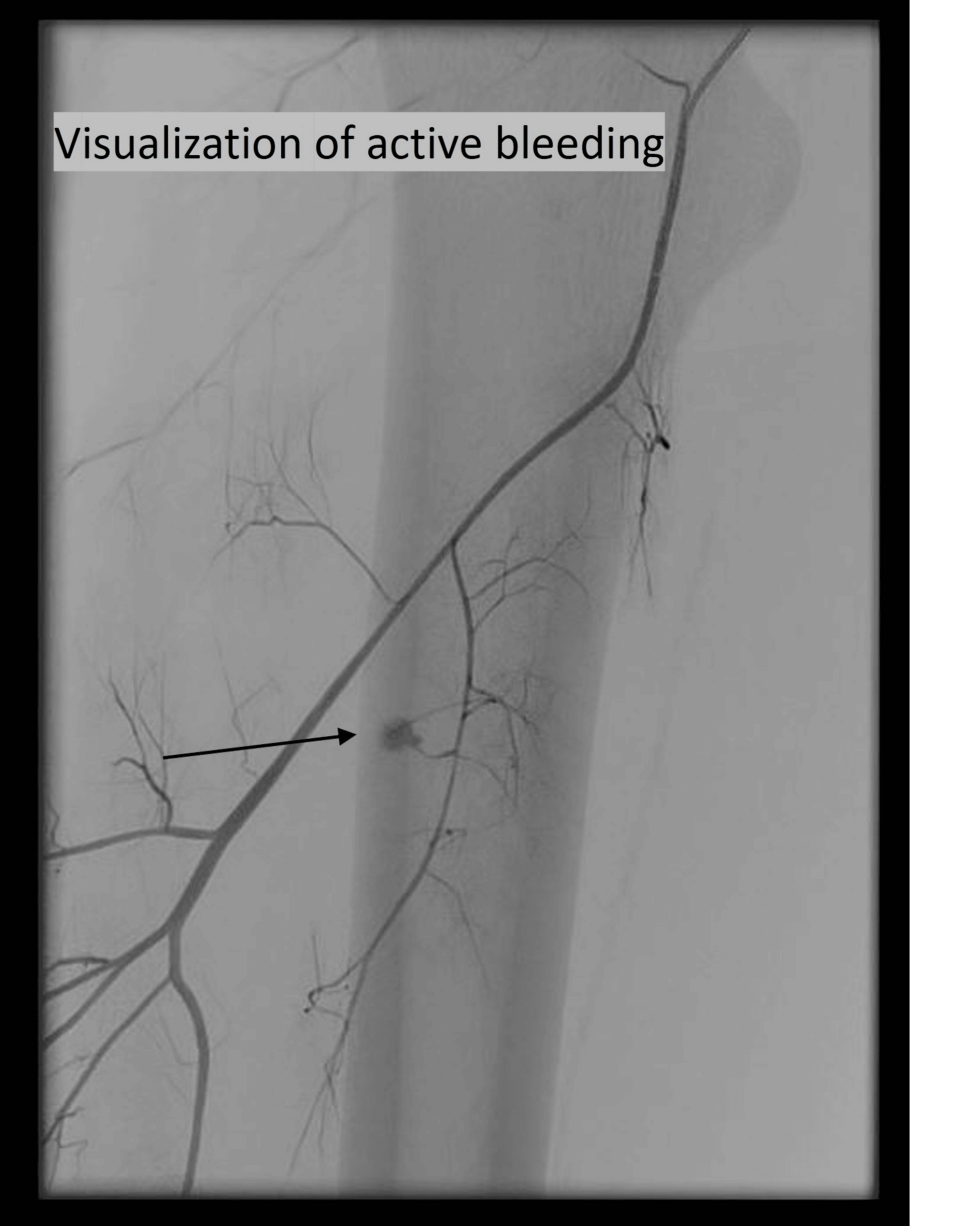
Selective digital subtraction angiography of the right profunda femoris artery demonstrates focal contrast extravasation from a distal branch, consistent with active hemorrhage (arrow)

Upon admission to our emergency department, progressive swelling of the right thigh compared to the initial examination was observed, with a circumference difference of 6 cm between the right and left thighs. The hemoglobin level decreased significantly from 9.4 mmol/L to 8.1 mmol/L within one hour. The patient reported moderate pain under analgesic treatment. Based on these findings, the decision was made to proceed with coil embolization of the bleeding profunda femoris artery branch (Figure 2).

**Figure 3:**
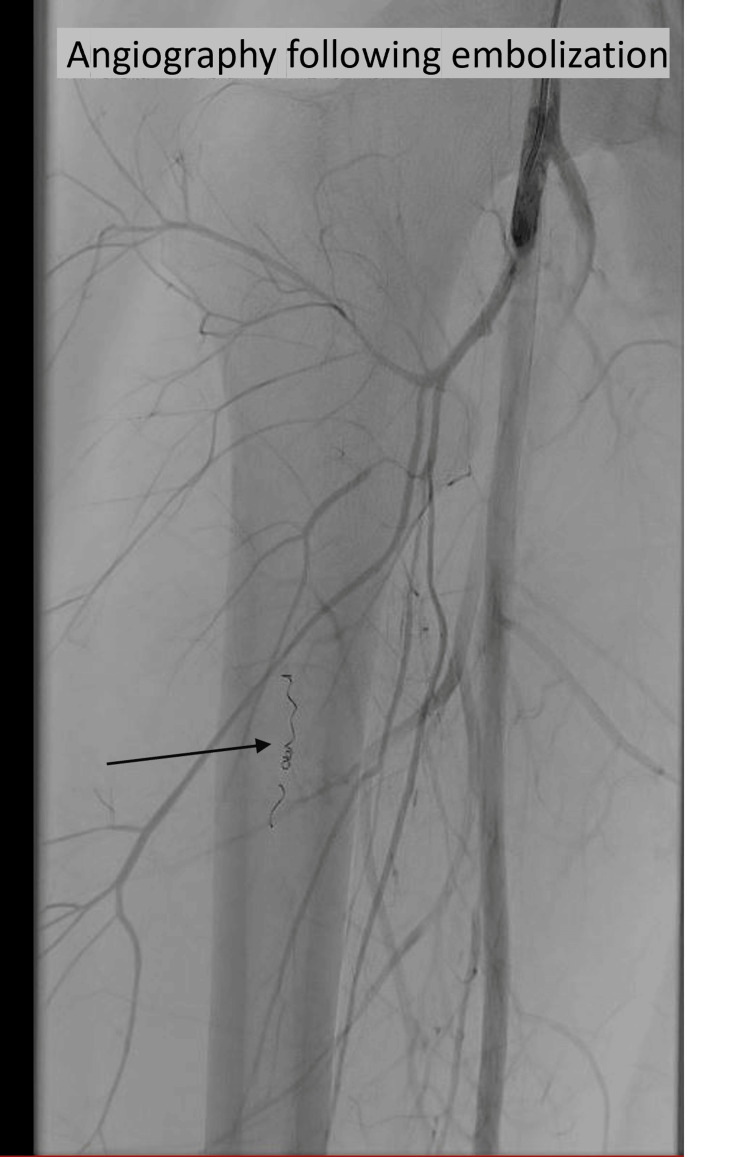
Post-interventional angiography following embolization with coils in place

The procedure was successfully performed by radiologists using contralateral femoral artery access and intervention in a crossover technique under sedation (Figure 3). Following embolization, the patient was admitted to the pediatric intensive care unit for close monitoring. Pain was assessed every two hours using a visual pain rating scale. Thigh circumference was assessed on both sides, measured once 10 cm above the superior pole of the patella and once at the midpoint between the inguinal skin fold and the superior pole of the patella. The measurement sites were marked with a pen to allow reproducible measurements. Additional monitoring parameters included clinical inspection for tense or shiny skin, assessment of motor and sensory function, and capillary refill time. Gray-scale ultrasound was used to evaluate the hematoma size and the striation of the muscle in this area.

Initial laboratory investigations showed elevated serum creatine kinase (CK) levels, approximately seven times above the upper limit of normal, and a serum myoglobin level of 355 µg/L (reference range: 0-96 µg/L). Serum creatinine, blood urea nitrogen, cystatin C, alanine aminotransferase (ALT), aspartate aminotransferase (AST), and white blood cell count remained within normal limits during both initial and follow-up evaluations.

As no clinical signs of CS were observed, invasive compartment pressure measurement was deemed unnecessary.

After 12 hours of bed rest, active mobilization was started, first partial, then full weight-bearing in accordance with the pain. To achieve early mobilization, the patient was on scheduled dosing of metamizole, administered every six hours and an additional Ibuprofen and piritramide medication on an as-needed basis. On day four after admission, the patient was discharged with ibuprofen prescribed as needed and instructions for weight-bearing mobilization according to pain tolerance.

## Discussion

In the present case, the assessment for a developing CS was primarily based on careful clinical examination. Characteristic clinical hallmarks include worsening pain at rest with increasing analgesic requirements, pain out of proportion to the injury, and exacerbation of pain during passive muscle stretching. Each of the three compartments of the thigh can be assessed separately. In the most affected anterior compartment, including the sartorius muscle and the quadriceps femoris, passive muscle stretch can be forced by knee flexion. Hip abduction tests the medial compartment including M. adductor longus, M. adductor magnus and brevis as well as the gracilis muscle. M. biceps femoris, M. semimembranosus and M. semitendinosus are located in the posterior compartment and could be tested by passive knee extension [4,5].

In the absence of clinical signs suggestive of CS, invasive compartment pressure measurement was not deemed necessary in our case. In routine clinical practice, CS is predominantly diagnosed based on clinical findings, without an increased risk of diagnostic delay. This approach is supported by a review by Ojike et al. (2010), which analyzed nine studies including 89 patients, as well as by the study conducted by Al-Dadah et al. (2008) [1,6]. In the latter study, two cohorts of patients with tibial diaphyseal fractures (each comprising 109 patients) were compared: one group was monitored using continuous anterior compartment pressure measurement, while the other was assessed through serial clinical examinations [6].

Furthermore, compartment pressure measurement remains controversial, as its accuracy depends on technical expertise and patient-related factors. It is therefore predominantly utilized in patients who are sedated or mechanically ventilated [7]. One of the major challenges in establishing reliable clinical or objective diagnostic criteria is that CS is not solely defined by increased pressure within a dermato-fascial compartment but also by a complex pathophysiological cascade. This cascade includes impaired venous outflow, increased interstitial pressure, tissue ischemia, capillary leakage, and rhabdomyolysis, ultimately perpetuating a self-sustaining vicious cycle [5].

In general, if the absolute pressure within a muscle compartment exceeds 30 mm Hg, or if the differential pressure, the difference between diastolic blood pressure and measured compartment pressure, is less than 30 mm Hg, dermato-fasciotomy of all compartments of the affected limb is indicated [8-10]. In CS, a self‑perpetuating cycle of rising pressure, impaired perfusion, and the accumulation of harmful metabolites leads to progressive muscle injury, potentially resulting in muscle necrosis. Nerve damage can cause severe consequences, including paralysis and sensory loss [5]. Further risk arises from muscle necrosis and rhabdomyolysis, as the release of myoglobin may lead to acute kidney injury [5]. In the long term, CS may also result in complex regional pain syndrome [11]. Therefore, delayed recognition and treatment of CS can lead to irreversible functional impairment. In patients presenting with paralysis at the time of diagnosis, only a small minority achieved meaningful recovery [2,3].

Adequate pain management is essential for patient recovery and early mobilization and is a fundamental obligation in modern medicine. However, concerns have been raised that certain analgesic strategies, including regional anesthesia, patient-controlled analgesia, or scheduled systemic medications, may mask early symptoms of developing CS. A systematic review of 28 case reports and case series with a total of 35 patients by Mar et al. (2009) found no association between analgesic technique and delayed diagnosis of CS, with epidural analgesia being the most commonly used method [12]. Similarly, the narrative review by Sonawane et al. (2022) advocates for consistent pain management, citing a lack of evidence that it delays the diagnosis of CS [5].

## Conclusions

In our case, the short duration of hospitalization and minimally invasive treatment were made possible by the effective collaboration between regional trauma centers and our tertiary care unit. This includes technical infrastructure for transmitting CT scans via the PACS, enabling immediate interdisciplinary planning while the patient is prepared for transfer. Interventional radiology facilitates minimally invasive management and plays a crucial role in controlling active bleeding, such as injuries to the circumflex femoral artery or branches of the profunda femoris artery after proximal femur fractures. Moreover, the complexity of injury patterns resulting from rollover accidents underscores the need for specialized multidisciplinary trauma care.
